# Cognitive impairment is associated with altered blood cell profiles in aggressive lymphoma

**DOI:** 10.1007/s00520-026-10317-6

**Published:** 2026-01-23

**Authors:** Delyse McCaffrey, Priscilla Gates, Haryana M. Dhillon, Carlene Wilson, Janette L. Vardy, Cynthia Shannon Weickert, Adam K. Walker

**Affiliations:** 1 Laboratory of ImmunoPsychiatry, Neuroscience Research Australia, Randwick, NSW Australia; 2 Discipline of Psychiatry and Mental Health, Faculty of Medicine, University of New South Wales, Sydney, Australia; 3 Cognitive Neuroscience Lab, School of Psychology, Deakin University, Burwood, VIC Australia; 4 Department of Clinical Haematology, Austin Health, Melbourne, VIC Australia; 5 Health Services Research, Peter MacCallum Cancer Centre, Melbourne, VIC Australia; 6 Faculty of Science, School of Psychology, Psycho-Oncology Cooperative Research Group, The University of Sydney, Sydney, NSW Australia; 7 Centre for Epidemiology and Biostatistics, Monash School of Population and Global Health, the University of Melbourne, Melbourne, VIC Australia; 8 Faculty of Medicine and Health, University of Sydney, Camperdown, NSW Australia; 9 Psycho-Oncology Cooperative Research Group, University of Sydney, Sydney, NSW Australia; 10 Concord Cancer Centre, Concord Repatriation General Hospital, Concord, NSW Australia; 11 Schizophrenia Research Laboratory, Neuroscience Research Australia, Randwick, NSW Australia; 12 Department of Neuroscience & Physiology, Upstate Medical University, Syracuse, NY USA

**Keywords:** Cognitive impairment, Lymphoma, Neutrophil-to-lymphocyte ratio, Platelet-to-lymphocyte ratio, Systemic immune-inflammation index, Inflammation, Anxiety, Depression, Executive function, Chemotherapy

## Abstract

**Purpose:**

Cognitive and psychological symptoms in neuropsychiatric disorders have been linked to blood cell parameters, including neutrophil-to-lymphocyte ratios (NLRs), systemic immune-inflammation indices (SIIs), and platelet-to-lymphocyte ratios (PLRs). It remains unclear whether cognitive impairments in haematological cancers are associated with biological vulnerabilities reflected in these parameters. We examined whether cognitive and psychological morbidity correlated with blood cell parameters before, during, and after chemotherapy in individuals with aggressive lymphoma.

**Methods:**

Neuropsychological testing and self-reported questionnaires were administered at diagnosis, mid-chemotherapy, and 6–8 weeks post-treatment (*n* = 30). Regression models assessed associations between cognition and blood cell parameters. Bootstrapped Pearson correlations examined relationships between NLRs, SIIs, PLRs, and psychological symptoms. To test specificity, similar analyses were conducted in healthy controls (*n* = 72).

**Results:**

In individuals with aggressive lymphoma, NLRs, SIIs, and PLRs correlated with impairments in inhibitory control, cognitive flexibility, delayed recall, and working memory across time points (*p* < 0.05). A disconnect emerged between these parameters and subjective self-reports. At diagnosis, lower NLRs, SIIs, and PLRs were associated with worse objective cognitive performance but better perceived cognition. Mid-chemotherapy, higher NLRs correlated with worse delayed recall but fewer reported depression and anxiety symptoms (*p* < 0.05). No significant associations were observed in healthy controls.

**Conclusion:**

Cognitive impairment was associated with blood cell parameters in individuals with aggressive lymphoma, indicating distinct biological patterns of dysfunction before, during, and after chemotherapy. The disconnect between objective neuropsychological performance and subjective self-reports reinforces the value of incorporating biomarkers into cognitive assessments in this population.

**Supplementary Information:**

The online version contains supplementary material available at 10.1007/s00520-026-10317-6.

## Introduction

Cognitive impairments such as executive dysfunction and verbal memory deficits, and psychological symptoms such as anxiety and depression, are common in individuals with non-central nervous system (non-CNS) cancers [1–4]. These symptoms often co-occur as a neuropsychological symptom cluster, which may also include fatigue, sleep disturbance, pain, and fear of cancer recurrence [5, 6]. The presence of such a cluster reflects the interrelated nature of these cognitive and psychological changes, which collectively exacerbate functional impairment, reduce quality of life, and impact patients’ ability to engage in daily activities and adhere to treatment regimens. A range of biological factors have been implicated in cognitive and psychological vulnerability in cancer populations, including lower baseline cognitive reserve and genetic variants (e.g., COMT, APOE), and emerging evidence suggests the gut microbiome may also modulate risk [7–11]. The central mechanism most often linked to these symptoms is immune system dysregulation resulting from both the cancer and anti-cancer treatments.Peripheral inflammatory signals released by non-CNS cancers or damaged cells can initiate neuroinflammatory processes within the brain through mechanisms such as oxidative stress, microglial activation, cytokine-mediated disruption of neurotransmitter systems, and altered neurogenesis [12–17]. These processes ultimately lead to changes in neural circuit function that compromise cognition and emotional regulation.

Several peripheral biomarkers have been investigated as potential indicators of cognitive and psychological vulnerability in cancer populations, including cytokines, neurotrophic factors and hormonal fluctuations. Proinflammatory cytokines such as interleukin (IL)-1β, IL-6, and tumor necrosis factor (TNF)-α have been linked to cognitive impairments and mood disturbances in some studies [18–21], but results are often inconsistent, with some reports showing no association or even contradictory patterns [22]. Brain-derived neurotrophic factor (BDNF), a key regulator of synaptic plasticity and neurogenesis, has also been examined, with lower circulating levels associated with worse cognitive outcomes and depression in certain cancer cohorts [23, 24]. However, similar to cytokines, findings for BDNF are variable across studies, likely due to small sample sizes, heterogeneous cancer types, differences in treatment regimens, timing of sample collection, and individual variability in systemic and CNS responses. Other explored biomarkers include markers of oxidative stress, cortisol, and hormonal factors, yet none have emerged as reliable, clinically actionable predictors [25–28]. These limitations highlight the need for accessible, robust, and reproducible biomarkers that can be integrated into routine clinical practice.

Blood cell ratios such as neutrophil-to-lymphocyte ratios (NLRs), systemic immune-inflammation indices (SIIs), and platelet-to-lymphocyte ratios (PLRs) shift in innate and adaptive immune activity, where elevated neutrophils and platelets, coupled with reduced lymphocytes, reflect heightened systemic inflammation. Such immune profiles may signal an increased propensity for neuroinflammatory signalling and vulnerability in neural systems governing cognition and mood. Similar patterns have been reported in neurodegenerative and neuropsychiatric disorders such as Alzheimer’s disease and schizophrenia, where comparable cognitive and psychological symptoms have been linked to alterations in blood cell parameters, including NLRs, SIIs, and PLRs [29–31]. If cognitive and psychological symptoms in aggressive lymphoma relate to immune changes mirrored in blood cell profiles, these parameters could function as accessible peripheral markers of immune-related vulnerabilities that contribute to neurocognitive dysfunction.

While chemotherapy and other treatments may contribute to cognitive decline, cancer itself can initiate early brain changes driven by immune dysregulation, which are further exacerbated by treatment and the psychosocial stress of diagnosis [13, 32, 33]. Although many survivors recover cognitive function, a subset of individuals experience persistent cognitive impairment long after treatment cessation [34, 35]. A major barrier to addressing cognitive symptoms in cancer populations is the difficulty in identifying individuals at high-risk for cognitive impairment, which may explain why cognitive and behavioural intervention trials that take a ‘one size fits all’ approach have yielded mixed results [36]. By accurately identifying those with cognitive morbidity, and pinpointing the specific cognitive domains affected, interventions could be tailored to individual needs and targeted to specific adverse outcomes. Leveraging existing clinical practices, such as routine blood tests, would avoid the need for comprehensive cognitive and psychological assessment in all patients, which lacks feasibility for most cancer clinics due to the immense cost and resource demands this would entail.

Individuals newly diagnosed with aggressive lymphoma are at risk of considerable cognitive impairment [37–40] and experience dynamic fluctuations in their circulating blood cell counts as a consequence of both the cancer and its treatment. This makes them an ideal population to explore associations between cognitive impairment and blood cell parameters. At diagnosis, people with advanced aggressive lymphoma often display lower NLRs, SIIs and PLRs due to increased lymphocyte proliferation in the bone marrow, which displaces the production of neutrophils and platelets. This can result in reduced circulating numbers of non-lymphocytes. Chemotherapy reduces overall white blood cell numbers, causing both neutropenia and lymphopenia [41]. Rituximab, a monoclonal antibody commonly used for lymphoma, targets CD20-positive B cells, and further depletes lymphocyte counts [42–44] while treatments like granulocyte colony-stimulating factor (G-CSF) stimulate neutrophil production [45]. Consequently, metrics such as NLRs, SIIs, and PLRs tend to be elevated in lymphoma patients during and immediately after active chemotherapy. Even as bone marrow recovers, ongoing treatment effects can keep these parameters elevated. This pronounced variability in blood cell parameters in aggressive lymphoma provides a unique opportunity to explore their relationships with cognitive and psychological symptoms, potentially uncovering associations that may be obscured in other patient populations where blood cell fluctuations are less pronounced.

In this study, we examined whether cognitive impairments in individuals with aggressive lymphoma are associated with distinct biological profiles, as indicated by fluctuations in blood cell parameters (NLRs, SIIs, PLRs), before, during and after chemotherapy. This work extends our previous study on the same cohort which examined average cognitive and self-report trajectories over time, without investigating biological contributors to individual differences in these symptoms [46]. On average, patients were significantly impaired on almost every neuropsychological test at each time point compared to healthy controls, highlighting the substantial cognitive burden in this population [47]. However, in the lymphoma cohort, we found that objective cognitive performance improved on average longitudinally, whereas psychological and self-reported cognitive symptoms worsened over time. Most notably, there was substantial individual variability in these trajectories that was unaccounted for when focusing on average trajectories alone. Relying on average changes alone may mask important heterogeneity and overlook individuals who experience greater cognitive or psychological impairment. The current study aims to help fill that knowledge gap by examining how fluctuations in blood cell parameters relate to cognitive and psychological outcomes at the individual level, providing new insight into the biological factors underlying symptom variability in aggressive lymphoma.

## Methods

### Study design and participants

This study comprised exploratory analyses using data from two sources: a longitudinal feasibility study focused on individuals newly diagnosed with aggressive lymphoma [46] and healthy controls from a longitudinal cohort study that evaluated cognitive function in colorectal cancer patients [1] (Table 1). Previously published reports [1, 46] provided a comprehensive overview of descriptive statistics, patient characteristics and cognitive outcomes. Statistical analyses were conducted using R version 3.6.1.

**Table 1:** Participant characteristics

	Patients *n* = 30		Controls *n* = 72	
Characteristics	*n*	%	*n*	%
*Age at enrolment (years)*				
Mean (SD)	57 (17)		56 (11)	
Median (IQR)	61 (50–69)		58 (46–63)	
Range	18–78		26–75	
*Sex*				
Male	16	53	31	43
Female	14	47	41	57
*Years of formal education*				
Mean (SD)	13 (2)		14 (3)	
Median (IQR)	13 (12–14)		15 (11–15)	
Range	7–18		6–20	
*Diagnosis*				
Diffuse large B cell lymphoma	20	67		
Grade 3B follicular lymphoma	1	3		
Hodgkin Lymphoma	4	13		
Mantle cell lymphoma	1	3		
T-cell lymphoma	3	10		
Primary mediastinal B-cell lymphoma	1	3		
*Chemotherapy regimen*				
ABVD × 6	3	10		
CHOP × 6	2	7		
Esc-BEACOPP × 4	1	3		
Mini R-CHOP × 6	2	7		
R-CHOP × 2	1	3		
R-CHOP × 3	2	7		
R-CHOP × 4	3	10		
R-CHOP × 6	10	33		
R-CHOP & HD MTX × 2	1	3		
R-CHOP & R × 2	4	13		
R-CHOP/R-DHAP × 3	1	3		
*Chemotherapy treatment (days)*				
Mean (SD)	102 (34)			
Median (IQR)	105 (105–114)			
Range	21–116			

*ABVD* adriamycin, bleomycin, vinblastine, dacarbazine, *R-CHOP* rituximab, cyclophosphamide, doxorubicin, vincristine, prednisolone, *Esc-BEACOPP* bleomycin, etoposide, doxorubicin, cyclophosphamide, vincristine, procarbazine, prednisolone, *HD MTX* high-dose methotrexate, *R-DHAP* rituximab, dexamethasone, cytarabine, cisplatin

People with newly diagnosed lymphoma (*n* = 30) were aged 18 to 78, scheduled for standard curative combination chemotherapy, proficient in English, and rated with Eastern Cooperative Oncology Group (ECOG) Performance Status of 0 to 2. Participants were excluded if they exhibited lymphomatous CNS involvement, had undergone or were planned to undergo cranial radiation therapy, had a life expectancy of less than 12 months, suffered from medical conditions that could interfere with treatment adherence or prolong hospital stays, or had a history of substance misuse or poorly managed psychiatric disorders.

Healthy controls (*n* = 72) were adults aged 18 to 75. Exclusion criteria included a prior history of malignancy, conditions that could affect cognitive function, major psychiatric illnesses, alcohol misuse, abnormal haematological, renal, or liver function, or inadequate English proficiency. Characteristics of the control group have been detailed in previous studies [1].

### Recruitment procedures

Participants with newly diagnosed aggressive lymphoma were enrolled from a specialised haematology department at a cancer centre in Melbourne, Australia. Eligible individuals were approached by a research nurse and invited to join the study. Ethical approval has been granted by Austin Health Human Rights Ethics Committee (HREC) in Victoria, Australia. Healthy controls were recruited from community members and hospital visitors at six different hospitals in Sydney, Australia. The study had research ethics board approval at each institution. All participants provided written informed consent. The original studies were conducted in compliance with the principles of the Declaration of Helsinki (2013) and the principles of Good Clinical Practice and the Australian National Statement on Ethical Conduct in Human Research.

#### Data collection

Data collection for people with aggressive lymphoma occurred at three time points: at diagnosis, mid-chemotherapy cycle, and 6–8 weeks post-chemotherapy. A summary of the cognitive tests, and the domains evaluated can be found in Table 2. Detailed descriptions of these assessments have been provided in earlier studies [48]. We followed the International Cognition and Cancer Task Force recommendations for using objective neuropsychological tests to assess cognitive function in people with cancer, which include a core set of assessments covering learning and memory, processing speed, executive function, and working memory [49].

**Table 2:** Neuropsychological tests and self-report measures assessed in people with aggressive lymphoma

Category	Measure	Domain assessed
*Neuropsychological test*	Verbal Learning Test-Revised (HVLT-R)	Learning and Memory
	Controlled Oral Word Association Test (COWAT)	Verbal Fluency and Semantic Function
	Stroop Colour and Word Test	Inhibitory Control
	Trail Making Test Part A (TMT A)	Speed of Information Processing/Attention
	Trail Making Test Part B (TMT B)	Cognitive Flexibility
	Digit Span (WAIS-R)	Attention and Working Memory
*Self-report measure*	QLQ-C30 Cognitive Functioning Scale (CF)	General Perceived Cognition
	FACT-Cog (Cognitive Function Subscale)	Memory, Attention, Mental Clarity
	Cognitive Failures Questionnaire (CFQ)	Attention and Memory
	FACT-G	Quality of Life
	FACIT-F (Fatigue Subscale)	Fatigue
	PROMIS Depression 8b	Depression
	PROMIS Anxiety 7a	Anxiety

Full blood counts, obtained as part of routine care, were used to calculate blood cell parameters: NLRs, SIIs, and PLRs. NLRs were derived by dividing the absolute neutrophil count by the absolute lymphocyte count, SIIs were calculated using the formula: (platelet count × neutrophil count) / lymphocyte count, and PLRs were obtained by dividing the absolute platelet count by the absolute lymphocyte count.

**Fig. 1:**
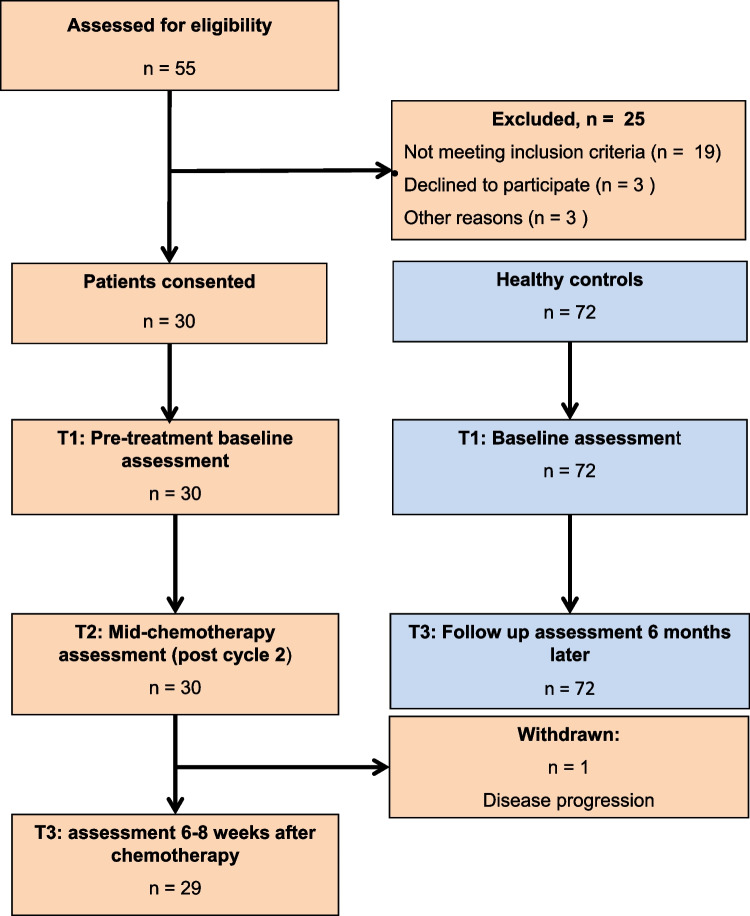
Participant flowchart. Of the 55 patients initially screened, 33 met the inclusion criteria and were invited to participate, with 30 providing consent. Ineligibility (*n* = 25) was primarily due to comorbidities affecting compliance with assessments (*n* = 11) or receiving treatment at external facilities (*n* = 3). Additionally, 3 patients declined to participate, and 3 were too distressed or overwhelmed by their diagnosis or treatment to enrol. The study ultimately included 30 treatment-naive patients with aggressive lymphoma undergoing standard chemotherapy. A healthy control group *(n* = 72) was also included for baseline and 6-month follow-up assessments for comparison

Healthy controls underwent a baseline assessment and a follow-up evaluation six months later, which aligned with diagnosis and post-chemotherapy of the lymphoma cohort. Neuropsychological assessments of cognitive performance of healthy controls included Hopkins Verbal Learning Test-Revised (HVLT-R), Trail Making Test (TMT) Part A and B, and the Wechsler Adult Intelligence Scale-Revised (WAIS-R). Subjective measures included the FACT-Cog, FACT-G, and the FACIT-fatigue (F) subscale. NLRs, PLRs, and SIIs were also assessed at each time point. A participant flowchart is provided in Fig. 1.

### Statistical analyses

#### Univariable and multivariable linear regression models

Univariable and forced-entry multivariable linear regression analyses were performed to assess whether blood cell parameters were associated with changes in either subjective and objective cognitive outcomes. Due to significant multicollinearity among the blood cell parameters (*p* < 0.01, data not shown), which affected the model's ability to identify meaningful predictors, cognitive outcomes were regressed on each blood cell parameter (PLR, NLR, or SII). Univariable analyses were used to screen potential predictors, and those with *p* < 0.1 were included in the final multivariable model. The supplementary material presents the full univariable analyses for the predictors carried forward to the multivariable models, serving as a guide for which variables were entered into each model. Covariates such as age, gender, and education were included in the multivariable models only if they were significant predictors of the specific blood cell parameter, in order to avoid overfitting by including non-significant variables. Disease stage, symptom classification (A or B) and chemotherapy regime did not explain significant variance in blood cell parameters at any time point and were not included in the multivariable models. The normality and homoscedasticity of standardised residuals were checked using Q-Q plots and scatterplots. If residuals were not normally distributed or exhibited unequal variances, the dependent variable was log-transformed. One outlier in blood cell parameters was identified at diagnosis (standardised residuals < -3 or > 3), and was excluded from the analysis at this time point. Variance inflation factors (VIFs) were used to detect multicollinearity, and any variables with VIFs exceeding 5 were removed from the model. Predictor variables were considered statistically significant if *p* < 0.05 in the final multivariable model.

#### Bootstrapped Pearson correlation coefficients

Three participants did not complete the questionnaires for anxiety and depression at one or more time points, therefore, Pearson correlation coefficients were used to assess the relationships between blood cell parameters (NLRs, SIIs and PLRs) and these psychological variables at each time point. Bias-corrected and accelerated (BCa) bootstrap confidence intervals were calculated based on 1000 bootstrapped samples to provide more accurate estimates.

#### Robust mixed-effects models

Given the non-normal distribution of standardised residuals, robust linear mixed-effects models were employed to determine the trajectory of average blood cell counts in people with aggressive lymphoma across time. Mixed models included fixed effects for time, and to accommodate repeated measures and the nested structure of the data, random intercepts were incorporated for each participant. To meet the requirements for subsequent pairwise comparisons, raw data were determined to be normally distributed using the Shapiro–Wilk and Kolmogorov–Smirnov tests, as well as normal Q-Q plots. If a significant main effect of time was detected, pairwise comparisons were employed to determine which time points were different.

#### R packages used

All statistical analyses, unless otherwise specified, were conducted using R (version 4.3.2). Bootstrapped analyses employed the boot package [50]. The “robustlmm” package in R was used to fit robust linear mixed-effects models [51] and pairwise post-hoc comparisons were performed using the “emmeans” package [52]. Diagnostic Q-Q plots and scatterplots were created with the ggplot2 package [53]. All figures were prepared using both GraphPad Prism (version 9.2) and R (version 4.3.2).

## Results

### Average blood cell counts in individuals with aggressive lymphoma across all three time points

We first characterised the changes in the average blood cell counts in individuals with aggressive lymphoma across all three time points. All three parameters showed a significant effect of time (estimate > -0.3139, t < -2.613, *p* < 0.014 for all) (see Supplementary Results). Pairwise comparisons indicated that chemotherapy was associated with a marked decrease in both neutrophil and lymphocyte levels, which remained low 6–8 weeks after chemotherapy (Supplementary Figure S1A-B). In contrast, platelet counts were comparable at diagnosis and mid-chemotherapy but were significantly reduced 6–8 weeks after treatment (Supplementary Fig. 1C).

### Specific cognitive impairments in people with aggressive lymphoma correlated with fluctuations in blood cell profiles before, during and after treatment

Next, we examined whether NLRs, SIIs and PLRs were correlated with cognitive and psychological outcomes at each time point.

#### Blood cell parameters correlated with perceived cognitive ability, executive functioning and fatigue in individuals with aggressive lymphoma at diagnosis

The univariable analyses identified perceived cognitive ability, perceived cognitive impairment (FACT-COG), fatigue total scores (FACIT-F), quality of life (FACT-G), TMT B T-scores, Stroop colour-word T-scores, and Stroop interference T-scores as significant correlates of one or more white blood cell parameter at diagnosis (all *p* < 0.1; Supplementary Tables S1-3), which advanced to forced entry multivariable linear regression modelling.

**Fig. 2:**
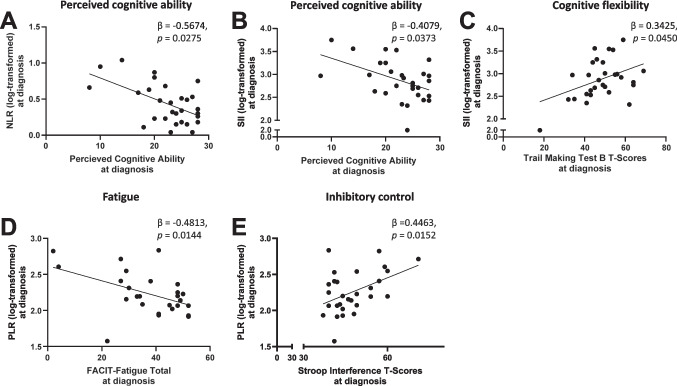
White blood cell ratios correlated with perceived cognitive ability, inhibitory control and fatigue in people with aggressive lymphoma at diagnosis. Linear regression analyses of significant relationships between blood cell parameters and self-reported/objective measures of cognitive functioning at diagnosis. NLRs (**A**) and SIIs (**B**) were negatively correlated with perceived cognitive ability, β = -0.5674, *p* = 0.0275; β = -0.4079, *p* = 0.0373. **C** SIIs were positively correlated to trail making B T-scores, β = 0.3425, *p* = 0.0450. **D** PLRs were negatively correlated with fatigue, β = -0.4813, *p* = 0.0144. ***(E)*** PLRs were positively correlated with Stroop interference T-scores, β = 0.4463, *p* = 0.0152. Abbreviations; PLR: Platelet-to-lymphocyte ratio, NLR: Neutrophil-to-lymphocyte ratio, SII: Systemic immune-inflammation index

When controlling for age, perceived cognitive ability was the only significant correlate of NLRs at diagnosis in the final multivariable model, explaining 17% of the total variance (Adjusted R^2^ = 0.1714) (Fig. 1A). In this model, perceived cognitive ability was negatively correlated with NLRs at diagnosis (Fig. 2A).

Both perceived cognitive ability and TMT B T-scores were significant correlates of SIIs in the final multivariable model, accounting for 26% of the total variance (Adjusted R^2^ = 0.2628) (Fig. 2B, C). Perceived cognitive ability was negatively correlated with SIIs, whereas TMT B T-scores showed a positive correlation at diagnosis (Fig. 2B, C).

Fatigue total scores (FACIT-F) were negatively correlated with PLRs at diagnosis, explaining 19% of the total variance (Adjusted R^2^ = 0.1924) (Fig. 2D). Stroop interference T-scores were positively correlated with PLRs at diagnosis, accounting for 17% of the total variance (Adjusted R^2^ = 0.1695) (Fig. 2E). Multivariable analyses for PLRs at diagnosis were not performed because FACIT-F fatigue scores and Stroop interference T-scores were correlated, preventing the model from effectively distinguishing their independent effects.

#### Neutrophil-to-lymphocyte ratios correlated with delayed recall in individuals with aggressive lymphoma mid-chemotherapy

In the univariable analyses, verbal learning and memory delayed recall T-scores were significant correlates of NLRs and SIIs mid-chemotherapy. Verbal learning and memory retention T-scores were also significant correlates of NLRs (Supplementary Tables S4-5). No significant correlates of PLRs mid-chemotherapy were identified.

**Fig. 3:**
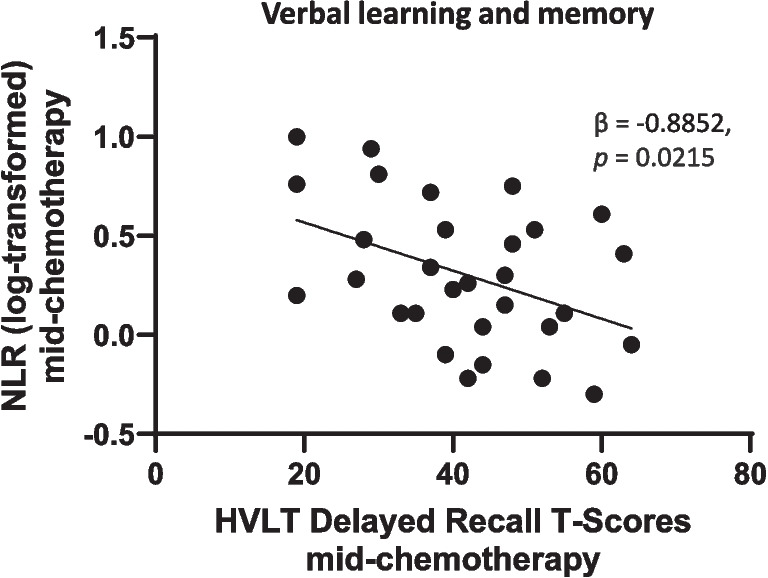
Neutrophil-to-lymphocyte ratios correlated with delayed recall in people with aggressive lymphoma mid-chemotherapy. Multivariable linear regression analyses of significant relationships between blood cell parameters and measures of cognitive functioning after two rounds of chemotherapy. NLR’s were negatively related to delayed recall, β = -0.8852, *p* = 0.0215. *Abbreviations; NLR: Neutrophil-to-lymphocyte ratio*

**Fig. 4:**
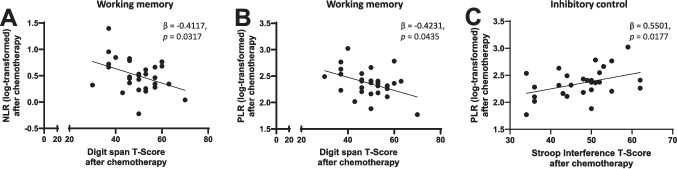
Blood cell parameters correlated with working memory and cognitive flexibility in people with aggressive lymphoma 6–8 weeks after chemotherapy. Multivariable linear regression analyses of significant relationships between blood cell parameters and objective measures of cognitive functioning 6–8 weeks after chemotherapy. **A** NLRs were negatively correlated with digit span T-scores, β = -0.4117, *p* = 0.0317.** B** PLRs were negatively correlated with digit span T-scores, β = -0.4231, *p* = 0.0435. **C** PLRs were positively correlated to Stroop interference T-scores, β = 0.5501, *p* = 0.0177. Abbreviations; PLR: Platelet-to-lymphocyte ratio, NLR: Neutrophil-to-lymphocyte ratio

In the multivariable models, delayed recall was the only significant correlate of NLR, explaining 19% of the total variance (Adjusted R^2^ = 0.1853) (Fig. 3), and was negatively correlated with NLRs (Fig. 4). Multivariable analyses were not conducted for SIIs because only one variable, delayed recall T-scores, reached *p* < 0.01 in the univariable analyses; however, it did not meet the threshold for statistical significance in the multivariable context.

#### Blood cell parameters were correlated with working memory and cognitive flexibility in individuals with aggressive lymphoma 6–8 weeks following chemotherapy

In the univariate analyses, digit span T-scores and Stroop colour T-scores were significant correlates of NLRs 6–8 weeks after chemotherapy. Total letter fluency T-scores, digit span T-scores, total recall T-scores, and recognition discrimination index T-scores were significant correlates of SIIs, while total letter fluency T-scores, total colour fluency T-scores, digit span T-scores, and Stroop interference T-scores were significant correlates of PLRs (Supplementary Tables S6-8).

After controlling for education, digit span T-scores were the only significant correlate of NLRs 6–8 weeks after chemotherapy in the final multivariable model, explaining 14% of the total variance (Adjusted R^2^ = 0.1445). Digit span T-scores were negatively correlated with NLRs at this time point (Fig. 4A).

After controlling for age, no significant correlates of SIIs 6–8 weeks after chemotherapy were identified in the final multivariable model.

Stroop interference T-scores and digit span T-scores were the only significant correlates of PLRs 6–8 weeks after chemotherapy in the final multivariable model, explaining 34% of the total variance (Adjusted R^2^ = 0.3426) (Fig. 4B, C). Digit span T-scores were negatively correlated with PLRs (Fig. 4B), whereas Stroop interference T-scores showed a positive correlation (Fig. 4C).

### Subjective reports of anxiety and depression were correlated with blood cell parameters mid-chemotherapy

Pearson correlation analyses revealed significant negative correlations between blood cell parameters and depression T-scores mid-chemotherapy. NLRs were negatively correlated with depression T-scores, with a 95% confidence interval (CI) ranging from -0.634 to -0.12 and a bootstrap standard error of 0.127 (Supplementary Table 13). Similarly, PLRs were negatively correlated with depression T-scores, with a 95% CI of -0.657 to -0.018 and a bootstrap standard error of 0.159 (Supplementary Table 13). The strongest correlation was observed for SIIs, which were negatively correlated with depression T-scores, with a 95% CI ranging from -0.706 to -0.167 and a bootstrap standard error of 0.115 (Supplementary Table 13). No significant correlations between blood cell parameters and depression T-scores were found at diagnosis or 6–8 weeks post-treatment.

A significant negative correlation between SIIs and anxiety T-scores was detected mid-chemotherapy (Supplementary Table 13). The 95% CI ranged from -0.603 to -0.138, with a bootstrap standard error of 0.122. No other significant correlations between blood cell parameters and anxiety T-scores were observed at any time point.

### Relationships between blood cell parameters and neuropsychological outcomes are specific to individuals with aggressive lymphoma

We tested whether the relationships between blood cell parameters and self-reported outcomes or objective cognitive tests were also evident in healthy controls. After adjusting for age and education where appropriate, no significant relationships at any time point were found for any of the variables assessed, including self-reported depression and anxiety measures (see Supplementary Results and Tables S9-12).

## Discussion

This study presents compelling evidence that cognitive impairments in people with aggressive lymphoma are associated with white blood cell ratios that vary at diagnosis, mid-chemotherapy and post-chemotherapy. NLRs, SIIs, and PLRs were related to four cognitive domains— inhibitory control, cognitive flexibility, delayed recall, and working memory —across all three time points. These findings highlight the potential for these blood cell parameters to inform personalised treatment plans by identifying individuals with cognitive impairment in specific domains before, during, and after treatment. In practical terms, these markers could be integrated into oncology practice by incorporating NLR, SII and PLR calculations from routine blood panels and using threshold values to flag individuals at risk. For example, a patient with lower NLRs, SIIs, and PLRs at diagnosis could be referred for early neurocognitive screening or a tailored program targeting inhibitory control, cognitive flexibility, and fatigue management, while patients showing higher NLRs and SIIs post-chemotherapy could receive targeted interventions such as memory strategy training or supportive cognitive rehabilitation to address deficits in working memory and delayed recall. Establishing validated cutoff scores for each marker at specific time points would be essential to guide these clinical decisions and standardise triage pathways across oncology clinics. We observed a discrepancy between how NLRs, SIIs, and PLRs relate to objective cognitive performance and subjective reports of cognitive and psychological morbidity, underscoring the complex interplay between biological and psychosocial factors. This divergence suggests that blood cell parameters may be better indicators of performance on neurocognitive tests than self-reported outcomes. Clinical assessments of cancer-related cognitive impairment often rely on an array of patient self-reports [54, 55]. However, our findings highlight the potential value of incorporating biological markers like blood cell profiles into cognitive evaluations to provide a more objective and reliable measure of cognitive function in patients.

Impairments in specific cognitive domains were correlated with fluctuations in NLRs, PLRs, and SIIs at diagnosis, mid-chemotherapy and post-treatment suggesting that neurocognitively vulnerable patients exhibit distinct biological phenotypes at different time points (Supplementary Fig. 2). Previous studies have shown that individuals with aggressive lymphoma have poorer performance on both objective and subjective cognitive measures compared to healthy controls at diagnosis and 6–8 weeks after chemotherapy [37, 38, 56]. Our study extends these findings by showing that individuals with lower NLRs, SIIs and PLRs at diagnosis exhibited reduced inhibitory control and less cognitive flexibility. At diagnosis, prior to any treatments, the hyperproliferation of lymphocytes, which are the denominator in NLR, SII, and PLR calculations, results in lower values for all of these parameters. This decrease reflects a higher cancer burden and is associated with greater cognitive impairment, suggesting that even before treatment begins, patients with more aggressive disease show greater cognitive dysfunction. At 6–8 weeks post-chemotherapy, the relationship between these blood cell parameters and cognitive impairment shifted with individuals with higher NLRs and SIIs having poorer working memory. This is likely because chemotherapy targets proliferating lymphocytes [41], leading to an increase in these parameters. Elevated NLRs, SIIs and PLRS can persist during the acute recovery phase as lymphocytes recover more slowly than neutrophils and platelets. Thus, at the latter timepoints, higher ratios likely reflect inflammation and immune recovery rather than cancer burden. Similar relationships between elevated PLRs and SIIs and poorer cognitive performance have been observed in long-term breast cancer survivors, decades after chemotherapy [35]. These findings highlight the need for personalised cognitive support and rehabilitation strategies, particularly for people with greater cancer burden, focusing on enhancing executive function and working memory.

It was more challenging to detect relationships between blood cell parameters and cognitive outcomes mid-chemotherapy. In contrast to other time points only delayed recall was associated with changes in NLRs, while no significant relationships were found between SIIs, PLRs and either objective or subjective cognitive outcomes. This is not unexpected given the heterogeneity of our cohort, which included patients receiving various chemotherapy regimens that can differentially impact blood cell profiles. Nevertheless, NLRs were most consistently related to cognitive impairment in people with aggressive lymphoma during active chemotherapy. In a larger cohort, additional associations between blood cell parameters and cognitive/psychological outcomes may be detected, and subgroup analyses by chemotherapy regimen or rituximab exposure could help clarify how specific treatments influence these relationships.

The discrepancy between the relationships of blood cell parameters and both objective cognition and subjective self-reports suggests that relying only on subjective reports is insufficient for identifying all individuals with measurable cognitive deficits. Patient-reported outcome measures capture the individual’s perceived experience of cognitive function and emotional well-being, whereas neuropsychological tests objectively measure performance in specific cognitive domains; the two do not always align. At diagnosis, individuals with higher NLRs, SIIs, and PLRs demonstrated poorer performance on objective cognitive tests, despite paradoxically reporting better perceived cognitive abilities. A similar pattern was observed during active chemotherapy: those with higher NLRs, SIIs, and PLRs reported fewer symptoms of anxiety and depression, even though they exhibited poorer performance in delayed recall tasks. This disconnect may be influenced by mechanisms such as mood bias, anosognosia, fatigue, or expectation effects: anosognosia can lead some patients to underreport cognitive deficits despite objective impairment, whereas mood bias can cause individuals experiencing anxiety, depression, or fatigue to perceive their cognition as worse than it objectively is. This is consistent with other studies in ovarian and breast cancer patients showing that neuropsychological impairment is not directly associated with self-perceived cognitive deficits [57–59]. Even when patients report feeling well, objective cognitive deficits can persist and subtly disrupt daily activities, work performance, and decision-making, meaning that self-perception alone may underestimate the true impact on quality of life. Thus, our findings emphasise the opportunity to incorporate objective biological indices like NLRs, SIIs, and PLRs alongside subjective measures to help healthcare providers manage cognitive impairments by delivering interventions that address both the biological and psychosocial aspects of patient care.

### Limitations and future directions

It is possible that additional relationships between blood cell parameters and cognitive outcomes would have been detected using a larger sample size. It will be important for future studies to replicate these findings in a larger cohort to confirm the robustness of these associations. Although the correlations between blood cell parameters and objective cognitive performance suggest biologically-driven brain changes due to cancer and its treatments, the precise mechanisms underlying these cognitive deficits remain unclear. Many studies have implicated inflammation in cognitive and psychological morbidity in non-CNS cancers [60–63]. Future research could examine the relationship between blood cell parameters and neuropsychological outcomes alongside traditional markers of systemic and neuroinflammation, such as cytokines and translocator protein (TSPO), to help elucidate the mechanisms driving cognitive decline in people with aggressive lymphoma. Examining whether relationships between blood cell parameters and neuropsychological outcomes exist in other cancer types, including non-haematological cancers, is needed to better understand the extent to which these blood cell parameters may be applied in the clinic and for whom. Although the lymphoma cohort and healthy controls were well matched on age, education, and gender, they were recruited from different locations and at an earlier time, which may have introduced ascertainment bias due to unmeasured differences in health, lifestyle, or socioeconomic factors unrelated to lymphoma. While matching on key demographic variables helps mitigate some confounding, we acknowledge that recruiting from distinct settings remains an important limitation of our study.

## Conclusion

Here, we have shown that cognitive impairments in people with aggressive lymphoma are a biologically profiled phenomena that vary before, during and after chemotherapy. Our findings support the use of NLRs, PLRs and SIIs as accessible clinical markers for stage-sensitive or time sensitive identification, enabling personalised interventions and improved cognitive care pathways for cancer patients. The discrepancy between how NLRs, SIIs and PLRs relate to objective cognitive measures and subjective reports of cognitive and psychological symptoms underscore the benefits of using both biomarkers and subjective measures together to achieve a comprehensive assessment of cognitive health in people with aggressive lymphoma.

## Supplementary Information

Below is the link to the electronic supplementary material.

ESM 1 (DOCX 806 KB)

## Data Availability

Data will be made available upon request.
