# Supplementary material for: Cognitive impairment is associated with altered blood cell profiles in aggressive lymphoma
Source: Support Care Cancer. 2026 Jan 23;34(2):120. doi: 10.1007/s00520-026-10317-6 (PMC12827377; doi:10.1007/s00520-026-10317-6)
Supplement: Supplementary file 1 — (DOCX 806 KB) [file 520_2026_10317_MOESM1_ESM.docx]

SUPPLEMENTARY MATERIAL

**Supplementary Figure 1. Robust mixed models revealed significant changes in blood cell parameters at baseline (T1), mid-chemotherapy (T2), and 6–8 weeks post-chemotherapy (T3).** Robust mixed models revealed significant changes in blood cell parameters at baseline (T1), mid-chemotherapy (T2), and 6–8 weeks post-chemotherapy (T3). ***(A)*** Neutrophil counts exhibited a significant main effect of time (estimate = -0.64, t = -2.95, *p* = 0.004), with pairwise comparisons indicating a significant reduction during chemotherapy compared to baseline, which remained low post-chemotherapy. ***(B)*** Lymphocyte counts also demonstrated a significant main effect of time (estimate = -0.31, t = -4.59, *p* < 0.001), showing a marked decrease during chemotherapy relative to baseline that persisted 6–8 weeks after treatment. ***(C)*** Platelet counts showed a significant main effect of time (estimate = -21.79, t = -2.61, *p* = 0.014); however, pairwise comparisons revealed that counts remained stable during chemotherapy compared to baseline but significantly declined post-chemotherapy. + SEM, n = 30*.*  *p* < 0.01, ****p* < 0.001, *****p* < 0.0001


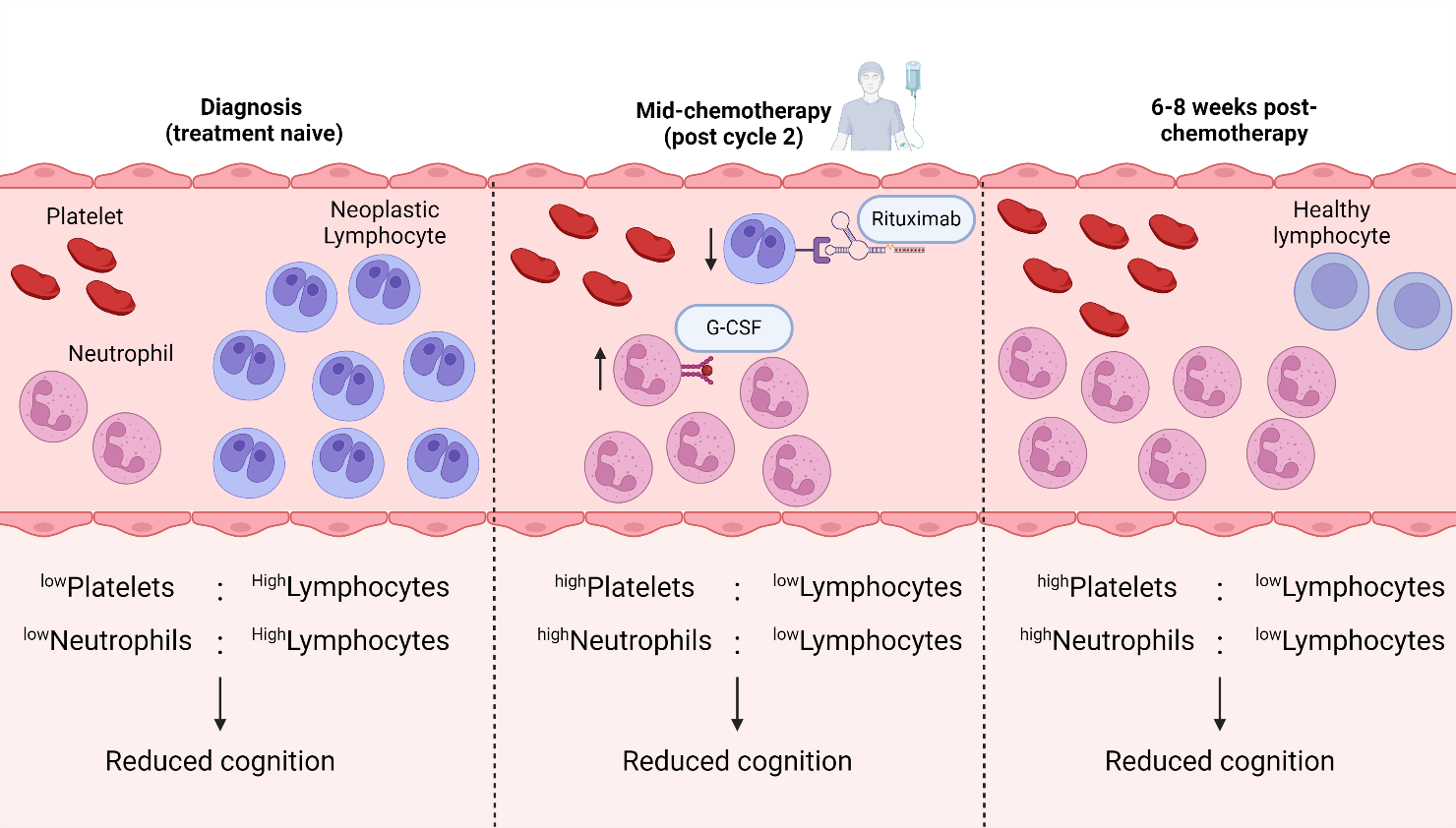


**Supplementary Figure 2. A schematic illustrating the association between cognitive impairment and blood cell profiles across the treatment trajectory in aggressive lymphoma.** At diagnosis (treatment-naïve), patients with a higher cancer burden increased neoplastic lymphocyte proliferation. This leads to relatively low neutrophil and platelet counts, resulting in low neutrophil-to-lymphocyte and platelet-to-lymphocyte ratios, patterns that are associated with poorer cognitive performance. By mid-chemotherapy (after cycle 2), treatment with agents such as rituximab and supportive therapies like granulocyte-colony stimulating factor (G-CSF) begin to suppress lymphocytes and increase neutrophils and platelets. At 6–8 weeks post-chemotherapy, neutrophils and platelets continue to recover, while lymphocyte counts remain low, leading to persistently high neutrophil-to-lymphocyte and platelet-to-lymphocyte ratios. Thus, at the latter timepoints, higher ratios likely reflect inflammation and immune recovery rather than cancer burden which is associated with reduced neurocognitive performance. *Created with BioRender.com.*

**Table S1. Significant univariable analyses between neutrophil-to-lymphocyte ratios and cognitive outcomes in people with aggressive lymphoma at diagnosis**

| **Measure** | **Coefficient**  **(b) (95% CI)** | **SE** | **t** | **F** | **Adjusted R^2^** | **p-value** |
| --- | --- | --- | --- | --- | --- | --- |
|  | **Subjective measures** | | | | | |
| **FACT-Cog** |  |  |  |  |  |  |
| Perceived cognitive impairment | -0.066 | 0.038 | -1.800 | 3.239 | 0.072 | 0.0827 |
| Perceived cognitive ability | -0.136 | 0.071 | -1.904 | 3.625 | 0.083 | 0.0670 |
| Impact on Quality of life | -0.182 | 0.094 | -1.944 | 3.777 | 0.087 | 0.0621 |
| **FACT-G** |  |  |  |  |  |  |
| Total score | -0.061 | 0.030 | -2.037 | 4.148 | 0.104 | 0.0520 |
| **FACIT-F** |  |  |  |  |  |  |
| Total score | -0.109 | 0.032 | -3.37 | 11.41 | 0.286 | 0.0024 |
|  | **Objective measures** | | | | | |
| **Stroop Colour and Word Test** |  |  |  |  |  |  |
| Word/colour | 0.097 | 0.042 | 2.318 | 5.375 | 0.131 | 0.0279 |
| Interference word/colour | 0.161 | 0.043 | 3.723 | 13.86 | 0.307 | 0.0009 |

**Table S2. Significant univariable analyses between systemic immune-inflammation indices and cognitive outcomes in people with aggressive lymphoma at diagnosis**

| **Measure** | **Coefficient**  **(b) (95% CI)** | **SE** | | | **t** | | **F** | | **Adjusted R^2^** | **p-value** |  |
| --- | --- | --- | --- | --- | --- | --- | --- | --- | --- | --- | --- |
|  | **Subjective measures** | | | | | | | | | |  |
| **FACT-Cog** |  |  | | |  | |  | |  |  |  |
| Perceived cognitive ability | -71.770 | 36.790 | | | -1.951 | | 3.807 | | 0.088 | 0.0611 |  |
| Impact on Quality of life | -84.620 | 49.150 | | | -1.722 | | 3.777 | | 0.087 | 0.0621 |  |
| **FACIT-F** |  |  | | |  | |  | |  |  |  |
| Total score | -46.690 | 18.030 | | | -2.589 | | 2.964 | | 0.063 | 0.0961 |  |
|  | **Objective measures** | | | | | | | | | |  |
| **Stroop Colour and Word Test** |  |  | | |  | |  | |  |  |  |
| Word/colour | 56.230 | 21.140 | | | 2.660 | | 7.075 | | 0.173 | 0.0128 |  |
| Interference word/colour | 85.020 | 22.160 | | | 3.837 | | 14.720 | | 0.321 | 0.0006 |  |
| **Trail Making Test** | |  | |  |  | |  | |  | |  |
| Part B | | 28.340 | | 16.830 | 1.684 | | 2.836 | | 0.060 | | 0.1000 |

**Table S3. Significant univariable analyses between platelet-to-lymphocyte ratios and cognitive outcomes in people with aggressive lymphoma at diagnosis**

| **Measure** | **Coefficient**  **(b) (95% CI)** | **SE** | **t** | **F** | **Adjusted R^2^** | **p-value** |
| --- | --- | --- | --- | --- | --- | --- |
|  | **Subjective measures** | | | | | |
| **FACIT-F** |  |  |  |  |  |  |
| Total score | -6.961 | 2.212 | -3.146 | 9.899 | 0.255 | 0.0042 |
|  | **Objective measures** | | | | | |
| **Stroop Colour and Word Test** |  |  |  |  |  |  |
| Interference word/colour | 8.572 | 3.173 | 2.701 | 7.297 | 0.178 | 0.0116 |

**Table S4. Significant univariable analyses between neutrophil-to-lymphocyte ratios and cognitive outcomes in people with aggressive lymphoma mid-chemotherapy**

| **Measure** | **Coefficient**  **(b) (95% CI)** | **SE** | **t** | **F** | **Adjusted R^2^** | **p-value** |
| --- | --- | --- | --- | --- | --- | --- |
| **Hopkins Verbal Learning Test** |  |  |  |  |  |  |
| Delayed recall | -0.089 | 0.032 | -2.750 | 7.563 | 0.185 | 0.0103 |
| Retention | -0.055 | 0.031 | -1.744 | 3.042 | 0.066 | 0.0921 |

**Table S5. Significant univariable analyses between systemic immune-inflammation indices and cognitive outcomes in people with aggressive lymphoma mid-chemotherapy**

| **Measure** | **Coefficient**  **(b) (95% CI)** | **SE** | **t** | **F** | **Adjusted R^2^** | **p-value** |
| --- | --- | --- | --- | --- | --- | --- |
| **Hopkins Verbal Learning Test** |  |  |  |  |  |  |
| Delayed recall | -24.790 | 12.390 | -2.000 | 4.000 | 0.094 | 0.0553 |

**Table S6. Significant univariable analyses between neutrophil-to-lymphocyte ratios and cognitive outcomes in people with aggressive lymphoma 6-8 weeks after chemotherapy**

| **Measure** | **Coefficient**  **(b) (95% CI)** | **SE** | **t** | **F** | **Adjusted R^2^** | **p-value** |
| --- | --- | --- | --- | --- | --- | --- |
| **Stroop Colour and Word Test** |  |  |  |  |  |  |
| Colour | -0.173 | 0.0897 | -1.930 | 3.724 | 0.089 | 0.0642 |
| **Digit Span Wechsler Adult Intelligence Scale** |  |  |  |  |  |  |
| Digit span total | -0.191 | 0.087 | -2.179 | 4.750 | 0.118 | 0.0382 |

**Table S7. Significant univariable analyses between systemic immune-inflammation indices and cognitive outcomes in people with aggressive lymphoma 6-8 weeks after chemotherapy**

| **Measure** | **Coefficient**  **(b) (95% CI)** | **SE** | **t** | **F** | **Adjusted R^2^** | **p-value** |
| --- | --- | --- | --- | --- | --- | --- |
| **Hopkins Verbal Learning Test** |  |  |  |  |  |  |
| Total recall | -23.220 | 12.09 | -1.921 | 3.692 | 0.088 | 0.0653 |
| Recognition/discrimination index | -38.860 | 12.63 | -3.077 | 9.466 | 0.232 | 0.0048 |
| **Controlled Oral Word Association Test** |  |  |  |  |  |  |
| Total letter fluency | -27.37 | 11.28 | -2.427 | 5.893 | 0.149 | 0.0222 |
| **Digit Span Wechsler Adult Intelligence Scale** |  |  |  |  |  |  |
| Digit span total | -35.23 | 15.33 | -2.298 | 5.280 | 0.133 | 0.030 |

**Table S8. Significant univariable analyses between platelet-to-lymphocyte ratios and cognitive outcomes in people with aggressive lymphoma 6-8 weeks after chemotherapy**

| **Measure** | **Coefficient**  **(b) (95% CI)** | **SE** | **t** | **F** | **Adjusted R^2^** | **p-value** |
| --- | --- | --- | --- | --- | --- | --- |
| **Stroop Colour and Word Test** |  |  |  |  |  |  |
| Word/colour |  |  |  |  |  |  |
| Interference word/colour | 9.093 | 4.395 | 2.069 | 4.280 | 0.105 | 0.0483 |
| **Hopkins Verbal Learning Test** |  |  |  |  |  |  |
| Recognition/discrimination index | -7.285 | 3.455 | -2.109 | 4.447 | 0.110 | 0.0444 |
| **Controlled Oral Word Association Test** |  |  |  |  |  |  |
| Category fluency | -9.549 | 3.126 | -3.055 | 9.332 | 0.229 | 0.0050 |
| Total letter fluency | -7.465 | 2.815 | -2.651 | 7.269 | 0.177 | 0.0133 |
| **Digit Span Wechsler Adult Intelligence Scale** |  |  |  |  |  |  |
| Digit span total | -7.772 | 3.986 | -1.950 | 3.801 | 0.091 | 0.0617 |

| Parameter | r | *p* | Bias | 95% CI | Standard error |
| --- | --- | --- | --- | --- | --- |
| NLRs | -0.410 | 0.038 | -0.003 | -0.634 to -0.12 | 0.127 |
| PLRs | -0.407 | 0.039 | 0.003 | -0.657 to -0.018 | 0.159 |
| SIIs | -0.505 | 0.008 | -0.001 | -0.706 to -0.167 | 0.115 |

**Table S9. Significant correlations between blood cell parameters and depression T scores mid-chemotherapy**

**Table S10. Significant univariable analyses between neutrophil-to-lymphocyte ratios and cognitive outcomes in healthy controls at baseline**

| **Measure** | **Coefficient**  **(b) (95% CI)** | **SE** | **t** | **F** | **Adjusted R^2^** | **p-value** |
| --- | --- | --- | --- | --- | --- | --- |
| **Hopkins Verbal Learning Test** |  |  |  |  |  |  |
| Total recall | -0.026 | 0.012 | -2.188 | 4.789 | 0.051 | 0.0320 |
| Delayed recall | -0.022 | 0.012 | -1.893 | 3.583 | 0.036 | 0.0626 |
| Recognition/discrimination index | -0.035 | 0.020 | -1.759 | 3.094 | 0.029 | 0.0830 |

**Table S11. Significant univariable analyses between systemic immune-inflammation indices and cognitive outcomes in healthy controls at baseline**

| **Measure** | **Coefficient**  **(b) (95% CI)** | **SE** | **t** | **F** | **Adjusted R^2^** | **p-value** |
| --- | --- | --- | --- | --- | --- | --- |
| **Hopkins Verbal Learning Test** |  |  |  |  |  |  |
| Recognition/discrimination index | -9.643 | 5.320 | -1.759 | 3.286 | 0.0312 | 0.0742 |

**Table S12. Significant univariable analyses neutrophil-to-lymphocyte ratios and cognitive outcomes in healthy controls at follow up**

| **Measure** | **Coefficient**  **(b) (95% CI)** | **SE** | **t** | **F** | **Adjusted R^2^** | **p-value** |
| --- | --- | --- | --- | --- | --- | --- |
| **Hopkins Verbal Learning Test** |  |  |  |  |  |  |
| Recognition/discrimination index | -0.024 | 0.013 | -1.835 | 3.368 | 0.032 | 0.0707 |

**Table S13. Significant univariable analyses between systemic immune-inflammation indices and cognitive outcomes in healthy controls at follow up**

| **Measure** | **Coefficient**  **(b) (95% CI)** | **SE** | **t** | **F** | **Adjusted R^2^** | **p-value** |
| --- | --- | --- | --- | --- | --- | --- |
| **Trail Making Test** |  |  |  |  |  |  |
| Part B | 5.263 | 3.103 | 1.696 | 2.877 | 0.026 | 0.0943 |
